# Recombinant *Toxoplasma gondii* expressing OVA confers antigen-specific protection against *Eimeria falciformis*

**DOI:** 10.3389/fimmu.2026.1915667

**Published:** 2026-09-16

**Authors:** Yingying Sun, Yilin Yang, Fujie Xie, Zongyue Tao, Sixin Zhang, Xiaotong Yao, Tingyu Zhang, Xun Suo, Xianyong Liu

**Affiliations:** 1 State Key Laboratory of Veterinary Public Health and Safety; Key Laboratory of Animal Epidemiology and Zoonosis of Ministry of Agriculture, National Animal Protozoa Laboratory & College of Veterinary Medicine, China Agricultural University, Beijing, China; 2 College of Veterinary Medicine, Southwest University, Chongqing, China

**Keywords:** antigen-specific cellular immunity, C3d, *Eimeria*, live-vector vaccine, ovalbumin (OVA), protective immunity, *Toxoplasma gondii*

## Abstract

Coccidiosis, caused by protozoan parasites of the genus *Eimeria*, remains a major enteric disease that requires effective antigen-specific cellular immunity for protection. In this study, we evaluated genetically engineered *Toxoplasma gondii*, an apicomplexan parasite closely related to *Eimeria*, as a live-vector platform for antigen delivery and induction of protective immunity against *Eimeria* infection. Recombinant RHΔ*ku80* strains expressing ovalbumin (OVA) alone or fused with four tandem copies of complement fragment C3d (OVA-C3d_4_) were generated using a CRISPR/Cas9-mediated strategy. Stable transgene integration and expression were confirmed by PCR, immunofluorescence assay, western blotting, and whole-genome resequencing. Phenotypic characterization demonstrated that expression of OVA or OVA-C3d_4_ did not significantly affect parasite invasion, intracellular replication, plaque formation, or acute virulence in mice. Immunization with recombinant parasites induced robust antigen-specific cellular immune responses, as evidenced by increased frequencies of IFN-γ–secreting splenocytes following stimulation with OVA-derived CD4^+^ and CD8^+^ T-cell epitopes. Fusion with C3d_4_ modestly influenced immune responses by altering antibody isotype profiles in an inversely dose-dependent manner, although total antibody titers were not substantially increased. Protective efficacy was evaluated using challenge infection with OVA-expressing *Eimeria* falciformis. Mice immunized with RHΔ*ku80-OVA* or RHΔ*ku80-OVA-C3d_4_* exhibited significantly reduced oocyst shedding and alleviated intestinal pathology compared with unimmunized controls. However, no significant additional protective benefit was observed for the C3d_4_-containing recombinant strain compared with the OVA-expressing strain under the experimental conditions tested. Collectively, these findings demonstrate that recombinant *T. gondii* can serve as an effective live-vector platform for inducing antigen-specific cellular immunity and partial protection against OVA-expressing *E. falciformis* challenge. This study highlights the potential of protozoan-based vaccine platforms and supports a potential contribution of antigen-specific cellular immunity to protection against coccidiosis.

## 1 Introduction

Coccidiosis, caused by protozoan parasites of the genus *Eimeria*, is an enteric disease characterized by intestinal epithelial damage, impaired nutrient absorption, and reduced host performance (1). In murine models, *Eimeria* infection has been widely used to investigate host–parasite interactions and protective immunity (2). It is well established that resistance to *Eimeria* is largely antigen-specific and develops following primary exposure, enabling the host to limit parasite replication upon reinfection (3). Accumulating evidence indicates that cell-mediated immunity plays a central role in protection, particularly T cell–dependent responses associated with interferon-γ (IFN-γ) production (1, 4). Both CD4^+^ and CD8^+^ T cells contribute to parasite clearance, whereas disruption of IFN-γ signaling leads to increased parasite burden and oocyst shedding (4, 5). These findings highlight the importance of inducing robust antigen-specific cellular immune responses for effective control of coccidial infections.

Despite advances in vaccine development, current strategies against coccidiosis remain suboptimal. Live attenuated vaccines can induce protective immunity but are associated with limitations in safety, production, and stability (6). In contrast, subunit vaccines are safer but often fail to elicit sufficiently strong cellular immune responses required for protection against intracellular parasites (7). Therefore, there is a need for alternative vaccine platforms capable of efficiently delivering antigens while simultaneously inducing potent Th1-biased immune responses.

*Toxoplasma gondii (T. gondii)*, an obligate intracellular protozoan parasite belonging to the phylum Apicomplexa, has emerged as a promising candidate for live vector–based vaccine development. Due to its strong immunostimulatory capacity and ability to induce robust IFN-γ-associated cellular immune responses, *T. gondii* provides a favorable immunological environment for antigen presentation and T-cell activation (8). Compared with *Eimeria*, which is primarily considered a target pathogen and vaccine vector for coccidiosis, *T. gondii* provides a heterologous apicomplexan platform with a well-characterized capacity to stimulate systemic cellular immune responses. This feature makes *T. gondii* particularly useful for investigating whether antigen-specific immunity elicited by one apicomplexan parasite can provide protection against an antigen-matched *Eimeria* challenge (9). Advances in genetic manipulation have enabled the generation of recombinant *T. gondii* strains expressing heterologous antigens, making it a versatile platform for vaccine delivery (10). However, whether a *T. gondii–based* live vector can confer protection against *Eimeria* infection through antigen-specific immunity, rather than nonspecific immune activation, remains to be fully elucidated.

Ovalbumin (OVA) has been widely used as a model antigen to investigate antigen-specific immune responses, owing to the availability of well-characterized T-cell epitopes (11). Although recombinant *T. gondii* expressing OVA has previously been used to investigate antigen-specific immune responses, whether heterologous antigen delivery by a protozoan live vector can induce antigen-specific protection against *Eimeria* infection remains largely unexplored (12). To address this question, we employed an antigen-matched challenge model using OVA-expressing *Eimeria falciformis* (EfOVA), allowing the protective efficacy of OVA-specific immunity induced by recombinant *T. gondii* to be directly evaluated. In addition, complement fragment C3d has been explored as a molecular adjuvant that enhances antigen-specific humoral immune responses by engaging complement receptors on B cells (13). Fusion of C3d to target antigens has been shown to enhance immunogenicity in various vaccine systems (14, 15). However, its role in the context of highly immunogenic protozoan live vectors, particularly in infections where protection is primarily mediated by cellular immunity, remains unclear. Therefore, we further investigated whether fusion of OVA with the molecular adjuvant C3d_4_ could modulate the immune response and protective efficacy.

In this study, we generated recombinant *T. gondii* strains expressing OVA alone or fused to multiple copies of C3d and evaluated their immunogenicity and protective efficacy against challenge with OVA-expressing *Eimeria* in a murine model. By comparing these parasites with the parental RHΔ*ku80* strain, we assessed whether protection depends on antigen expression and whether incorporation of the C3d_4_-containing sequence into the recombinant construct modulates immune responses and protective outcomes. These findings provide insight into the potential of protozoan parasites as live vaccine vectors and emphasize the importance of antigen-specific cellular immunity in protection against coccidial infections.

## 2 Methods

### 2.1 Parasite, cell culture and animals

The type I *T. gondii* RHΔ*ku80* strain was maintained in our laboratory under standard conditions. This strain exhibits no significant differences from the wild-type RH strain in host cell invasion, intracellular replication and plaque formation. Additionally, it loses the capacity to form tissue cysts *in vivo* and fails to undergo the intestinal sexual development stage (16). Human foreskin fibroblast (HFF) cells were cultured in Dulbecco’s modified Eagle’s medium (DMEM; Macgene) supplemented with 10% fetal bovine serum (FBS; Sigma-Aldrich), 100 U/mL penicillin, and 100 μg/mL streptomycin. Infected cell cultures were incubated at 37 °C in a humidified atmosphere containing 5% CO_2_.

Female BALB/c mice (6 weeks old) and CD-1 outbred mice were obtained from Beijing HFK Bioscience Co., Ltd. and maintained under specific pathogen-free (SPF) conditions.

### 2.2 Generation and validation of transgenic parasite strain

The OVA and mouse C3d gene sequences were retrieved from NCBI (OVA: GenBank=AUD54738.1; C3d: GenBank=ABD66220.1). A flexible linker (GSGGGGSGGGGSGS) was appended to the N-terminus of the C3d sequence. Codon usage was optimized for the *T. gondii* RH strain using the RH codon frequency table from Kazusa (http://www.kazusa.or.jp/codon/) and the OPTIMIZER tool (http://genomes.urv.es/OPTIMIZER/). The optimized C3d sequence is provided in **Supplementary Data 1**. The optimized linker-C3d and OVA sequences were synthesized by Qingke Biotech Co., Ltd. The OVA sequence was further fused to the *T. gondii* GRA8 signal peptide at the N-terminus and a 3×HA tag at the C-terminus. The resulting expression cassette was cloned into the pUPRT-Tub vector, yielding pUPRT-Tub-OVA-3HA-mCherry. Four tandem copies of C3d were assembled by restriction-ligation and fused with pUPRT-Tub-OVA-3HA-mCherry, generating pUPRT-Tub-OVA-C3d_4_-3HA-mCherry.

For targeted genomic integration, freshly harvested RHΔ*ku80* tachyzoites were co-transfected with the respective plasmids and a CRISPR/Cas9 plasmid encoding a specific sgRNA targeting the UPRT locus. Recombinant parasites were selected using FUDR, and clonal lines were established by limiting dilution from single plaques. Correct integration and expression of the transgenes were confirmed by PCR, immunofluorescence assay (IFA), and western blotting. Primer sequences are listed in **Supplementary Table S1**. The resulting transgenic strains were designated RHΔ*ku80-OVA* and RHΔ*ku80-OVA-C3d_4_*.

### 2.3 Immunofluorescence assays

Human foreskin fibroblast (HFF) cells were seeded onto glass coverslips and cultured to confluence prior to infection with *T. gondii*. At designated time points post-infection, cells were fixed with 4% paraformaldehyde for 30 min, permeabilized with 1% Triton X-100 for 30 min, and blocked with 3% bovine serum albumin (BSA) for an additional 30 min.

The following primary and secondary antibodies were used: mouse anti-HA (1:500), and FITC-conjugated goat anti-mouse IgG (1:500). Primary and secondary antibodies were incubated sequentially for 1 h each at room temperature. For staining, coverslips were inverted onto 50 μL droplets of antibody solution prepared in blocking buffer and incubated in a humidified chamber. Between incubation steps, samples were washed three times with phosphate-buffered saline (PBS).

After staining, coverslips were mounted with ProLong Diamond Antifade Mountant (Thermo Fisher Scientific) and incubated at 37 °C to cure. Nuclei were counterstained with DAPI (Thermo Fisher).

### 2.4 Western blotting

Tachyzoites of RHΔ*ku80-OVA* and RHΔ*ku80-OVA-C3d_4_* strains were harvested and lysed in RIPA buffer (50 mM Tris-HCl, pH 7.4; 150 mM NaCl; 1% Triton X-100; 1% sodium deoxycholate; 0.1% SDS; Beyotime) supplemented with 1 mM phenylmethylsulfonyl fluoride (PMSF). Protein samples were separated by SDS–polyacrylamide gel electrophoresis (SDS–PAGE) and subsequently transferred onto nitrocellulose membranes.

Following transfer, the membranes were incubated with an anti-OVA primary antibody (1:2000) to detect recombinant OVA-containing proteins. GAPDH was used as a loading control and detected using an anti-GAPDH antibody (1:2000). After washing, the membranes were incubated with the corresponding HRP-conjugated secondary antibody(1:5000) for 1 h at room temperature. Immunoreactive bands were visualized using an enhanced chemiluminescence (ECL) detection system.

### 2.5 Whole-genome resequencing of the RHΔ*ku80-OVA-C3d_4_* strain

Genomic DNA was isolated from tachyzoites of RHΔ*ku80-OVA* and RHΔ*ku80-OVA-C3d_4_* strains using a CTAB-based extraction protocol. DNA quality and concentration were evaluated by agarose gel electrophoresis and Quant-iT PicoGreen fluorescence assay, respectively. The extracted DNA was subsequently fragmented by ultrasonication to generate appropriate insert sizes for library construction. Working DNA samples were stored at 4 °C, while long-term stocks were preserved at -20 °C.

Sequencing libraries were constructed using the Illumina TruSeq Nano DNA LT kit according to the manufacturer’s instructions, including end-repair, adapter ligation, PCR amplification, and library quality control. Prepared libraries were normalized, pooled, and subjected to high-throughput sequencing on an Illumina platform.

Raw sequencing data were processed to remove adaptor sequences and low-quality reads using AdapterRemoval and a sliding window quality-filtering approach. Clean reads were aligned to the reference genome using the Burrows–Wheeler Aligner (BWA), and duplicate reads were marked and removed using Picard tools. Local realignment around insertion–deletion regions was performed using IndelRealigner from the Genome Analysis Toolkit (GATK).

Variant detection, including single nucleotide polymorphisms (SNPs) and insertions/deletions (InDels), was carried out using GATK UnifiedGenotyper. Copy number variations (CNVs) were identified using CNVnator, and structural variations (SVs) were detected with BreakDancer. All identified variants were functionally annotated using ANNOVAR.

### 2.6 Invasion assay

Confluent HFF monolayers cultured in 12-well plates were infected with RHΔ*ku80*, RHΔ*ku80-OVA* and RHΔ*ku80-OVA-C3d_4_* tachyzoites at a multiplicity corresponding to 1 × 10^6^ parasites per well and incubated at 37 °C in 5% CO_2_ for 2 h to allow parasite invasion. Following incubation, the culture medium was removed, and cells were fixed with 4% paraformaldehyde for 30 min and blocked with 3% bovine serum albumin (BSA) for 30 min.

To distinguish extracellular parasites, samples were first incubated with mouse anti-SAG1 antibody, followed by Alexa Fluor 594–conjugated goat anti-mouse secondary antibody. After washing with phosphate-buffered saline (PBS), cells were permeabilized with 1% Triton X-100 for 30 min and subsequently incubated with rabbit anti-GAP45 antibody (1:500) to label total parasites. Alexa Fluor 488–conjugated goat anti-rabbit secondary antibody was then applied.

Under this differential staining protocol, extracellular tachyzoites were labeled with both red and green fluorescence, whereas intracellular parasites were stained only with green fluorescence (17).

### 2.7 Replication assay

HFF monolayers cultured in 12-well plates were infected with RHΔ*ku80*, RHΔ*ku80-OVA* and RHΔ*ku80-OVA-C3d_4_* tachyzoites at a density of 2 × 10^5^ parasites per well and incubated at 37 °C in a 5% CO_2_ atmosphere for 2 h to allow invasion. Following infection, monolayers were washed three times with phosphate-buffered saline (PBS) to remove extracellular parasites.

At 24 h post-infection, cells were fixed with 4% paraformaldehyde and stained with anti-GAP45 antibody (1:500) to visualize intracellular parasites. Parasite replication was evaluated by determining the number of tachyzoites within each parasitophorous vacuole. For each experimental condition, at least 100 vacuoles were counted. All experiments were performed in triplicate.

### 2.8 Plaque assay

Parasites were inoculated onto confluent HFF monolayers in 6-well plates and maintained in Dulbecco’s modified Eagle’s medium (DMEM) supplemented with 2% fetal bovine serum (FBS) for 7 days without disturbance. At the end of the incubation period, monolayers were gently washed with phosphate-buffered saline (PBS) and fixed with 100% ethanol for 10 min.

Plaques were visualized by staining with crystal violet solution (2 mL per well) for 5 min. The staining solution was prepared by dissolving crystal violet in ethanol and ammonium oxalate. After staining, wells were rinsed with water and air-dried before imaging.

### 2.9 Virulence assay

To assess parasite virulence, six-week-old female CD-1 mice were intraperitoneally injected with 100 tachyzoites (n = 5 per group). Survival was monitored daily for 14 days post-infection, and body weight was recorded throughout the observation period.

### 2.10 Passage and purification of *E. falciformis* expressing OVA

The OVA-expressing *E. falciformis* strain (EfOVA) used in this study was previously generated and maintained in our laboratory (unpublished). EfOVA was used as an antigen-matched challenge strain to evaluate whether OVA-specific immune responses induced by recombinant *T. gondii* could confer protection against *E. falciformis* infection.

Six-week-old BALB/c mice were used for the passage and purification of *Eimeria falciformis* expressing ovalbumin (EfOVA). Mice were orally gavaged with 100 EfOVA oocysts per mouse to establish infection and maintained under specific pathogen-free (SPF) conditions with free access to sterile drinking water and standard rodent chow throughout the experiment.

Mice were housed in isolators to prevent cross-contamination, and each mouse was placed in a cage with a mesh bottom to facilitate fecal collection. Prior to use, all cages, trays, drinking water, and rodent chow were sterilized by baking at 180 °C for 3 h to eliminate *Eimeria* oocysts and other potential pathogens.

At 8 days post-inoculation (dpi), cage trays were replaced to collect feces from infected mice for subsequent oocyst purification. The purified oocysts were stored in 2.5% K_2_Cr_2_O_7_ at 4 °C.

### 2.11 Immunizations and challenge experiment

#### 2.11.1 Immunization Protocol 1

Six-week-old female BALB/c mice were randomly assigned to six experimental groups (n = 15 per group). The immunization schedule and sampling timeline are shown in **Supplementary Table S2**.

The experimental groups included the unimmunized unchallenged control (UUC), unimmunized challenged control (UCC), OVA protein–immunized group, RHΔ*ku80* group, RHΔ*ku80-OVA* group, and RHΔ*ku80-OVA-C3d_4_* group.

On day 0, mice in the immunization groups received either 100 μg of OVA protein (Sigma, USA) emulsified with an equal volume of Complete Freund’s Adjuvant (CFA) or 100 tachyzoites of the indicated *T. gondii* strains via intraperitoneal injection. On day 14, mice received a booster immunization via the same route, with 100 μg of OVA protein emulsified with an equal volume of Incomplete Freund’s Adjuvant (ICFA) or 1000 tachyzoites of the corresponding *T. gondii* strains, respectively. To control acute parasite replication following *T. gondii* immunization, sulfadiazine sodium treatment was initiated 72 h after each parasite inoculation. Drinking water containing sulfadiazine sodium at a final concentration of 400 mg/L was provided for 7 consecutive days. An appropriate amount of sucrose was added to improve the palatability of the medicated drinking water.

On day 28, mice in all groups except the UUC group were orally challenged with 50 sporulated oocysts of OVA-expressing *Eimeria*.

#### 2.11.2 Immunization Protocol 2

Six-week-old female BALB/c mice were randomly divided into experimental groups (n = 10 per group). The immunization schedule is illustrated in **Supplementary Table S3**.

Mice were immunized intraperitoneally at day 0 and boosted at day 14 with either 100 μg of OVA protein or recombinant *T. gondii* strains expressing OVA or OVA-C3d_4_ at different doses. For the RHΔ*ku80-OVA* groups, mice received 10, 100, or 1000 tachyzoites per mouse. Similarly, mice in the RHΔ*ku80-OVA-C3d_4_* groups were inoculated with 10, 100, or 1000 tachyzoites per mouse. To control acute parasite replication, mice receiving recombinant *T. gondii* were treated with sulfadiazine sodium as described in the immunization protocol 1.

Blood samples were collected at designated time points (day 28) for subsequent analysis of antigen-specific antibody responses.

### 2.12 Detection of oocyst excretion

Following challenge with sporulated *E. falciformis* oocysts on day 28, fecal samples were collected daily from day 31 to day 42 of the experimental timeline (3–14 days post-challenge) to quantify oocyst shedding. Five mice from each group in immunization Protocol 1 were challenged with the same dose of sporulated oocysts to compare differences in oocyst excretion. Daily oocyst output was determined using a McMaster counting chamber, and the total daily oocyst output of the five mice was calculated to generate oocyst shedding curves.

### 2.13 ELISPOT

Five mice per group were selected post-second immunization, and splenocytes were isolated for ELISPOT to detect IFN-γ-secreting cells.

ELISPOT plates were pre-wetted with 15 μL pre-wetting solution for 1 min, then coated with 100 μL mouse IFN-γ capture antibody at 4 °C overnight. After discarding coating solution, plates were washed 5 times with sterile PBS and blocked with 200 μL 10% FBS RPMI 1640 at room temperature for 30–120 min. Mice were sacrificed by cervical dislocation and the body surface was disinfected with 75% ethanol. Spleens were isolated under biosafety cabinet, cleaned of connective tissues, and placed in pre-cooled 10% FBS RPMI 1640. Spleens were ground through an autoclaved 200-mesh copper screen to obtain cell suspensions (cut into pieces if needed). Cells were centrifuged (400 × g, 5 min); pellets were lysed with 5 mL RBC lysis buffer (RT, 5 min) and washed twice with PBS (400 × g, 5 min). Splenocytes were prepared as described above and adjusted to a concentration of 5 × 10^6^ cells/mL in RPMI 1640 medium supplemented with 10% FBS. A total of 100 μL of the cell suspension (5 × 10^5^ cells/well) was added to each well of the IFN-γ ELISPOT plate. Each sample was tested in duplicate. Synthetic OVA-derived peptides (>95% purity), including the CD4^+^ T-cell epitope (OVA 323-339) ISQAVHAAHAEINEAGR and the CD8^+^ T-cell epitope (OVA 257-264) SIINFEKL, were dissolved in DMSO to prepare 1 mg/mL stock solutions. For peptide stimulation, 3 μL of the corresponding peptide stock solution (3 μg peptide) was added to each well. The irrelevant peptide FILKSINE was included as a negative peptide control, and PBS and PMA/ionomycin were used as negative and positive controls, respectively. Plates were incubated at 37 °C in a 5% CO_2_ incubator for 24 h.

Following incubation, IFN-γ spots were developed according to the manufacturer’s instructions using biotinylated anti-mouse IFN-γ detection antibody, streptavidin-HRP, and AEC substrate. After air-drying, spots were quantified using an automated ELISPOT reader (AID, Germany) and the corresponding analysis software.

### 2.14 ELISA

Blood samples were collected from mice at 14 and 28 days after primary immunization. The samples were allowed to clot at 37 °C for 1 h, followed by incubation at 4 °C for 2 h. Serum was obtained by centrifugation at 5,000 rpm for 10 min and stored at -20 °C until further analysis.

Antigen-specific antibody responses were determined by enzyme-linked immunosorbent assay (ELISA). Specifically, total IgG antibodies against OVA were detected in the serum samples collected at 14 and 28 days after primary immunization under Immunization Protocol 1. In contrast, IgG1 and IgG2a antibodies against OVA were detected in the serum samples collected at 28 days after primary immunization under Immunization Protocol 2. All serum antibody detections were performed using the commercial Mouse (OVA-specific total IgG, IgG1, IgG2a) One Step ELISA Kit (LabEx Biotech, China).

### 2.15 Observation of intestinal lesions

On day 9 post-challenge, five mice from each group were euthanized by cervical dislocation. The abdominal cavity was opened, and the cecum was collected. The tissue was rinsed with pre-cooled phosphate-buffered saline (PBS), fixed in 4% paraformaldehyde for 24 h, and subjected to hematoxylin and eosin (H&E) staining for histopathological analysis. Histopathological scoring was performed in a blinded manner based on the severity of epithelial damage, tissue edema, and inflammatory cell infiltration. Lesions were graded on a scale from 0 to 5, where 0 represented normal tissue with no detectable lesions; 1 represented minimal lesions; 2 represented mild lesions; 3 represented moderate lesions; 4 represented marked lesions; and 5 represented severe lesions. Higher scores indicated more severe pathological damage.

### 2.16 Statistical analyses

All statistical analyses were performed using GraphPad Prism (version 8.3.0; GraphPad Software). Data are presented as mean ± standard deviation (SD). P-values are reported for the indicated statistical comparisons, with P < 0.05 considered statistically significant. Asterisks indicate the following levels of statistical significance: ns P > 0.05, *P < 0.05, **P < 0.01, ***P < 0.001, and ****P < 0.0001.

## 3 Results

### 3.1 OVA and OVA-C3d_4_ are successfully expressed in genetically engineered *T. gondii*

To generate recombinant *Toxoplasma gondii* strains stably expressing OVA or OVA-C3d_4_, expression cassettes were targeted to the UPRT locus via homologous recombination using a CRISPR/Cas9-mediated strategy (**Figures 1A, B**). Correct genomic integration of the transgenes was confirmed by diagnostic PCR using locus-specific primers (**Figure 1C**). To evaluate the genomic integration of the expression cassette in the transgenic strain, whole-genome resequencing was performed. High-quality reads were aligned to the reference genome, and sequencing depth across the genome was subsequently analyzed. As shown in **Figure 1D**, read coverage was generally uniform across most genomic regions, indicating high-quality sequencing and reliable mapping. Immunofluorescence assays demonstrated robust expression of the recombinant proteins in intracellular tachyzoites. The OVA signal was detected within the parasitophorous vacuole and showed a distribution pattern consistent with dense granule secretion (**Figure 1E**), indicating successful targeting and secretion of the expressed antigen. Notably, similar localization patterns were observed in both RHΔ*ku80-OVA* and RHΔ*ku80-OVA-C3d_4_* strains. Expression of the recombinant proteins was further validated at the protein level by Western blotting. Western blot analysis using an anti-OVA antibody detected specific OVA-reactive bands in the RHΔ*ku80-OVA* and RHΔ*ku80-OVA-C3d_4_* strains, whereas no corresponding signal was detected in the parental RHΔ*ku80* strain (**Figure 1F**). The apparent molecular mass of the OVA-reactive product in the RHΔ*ku80-OVA-C3d_4_* strain was lower than the calculated theoretical molecular weight of the full-length fusion protein.

**Figure 1:**
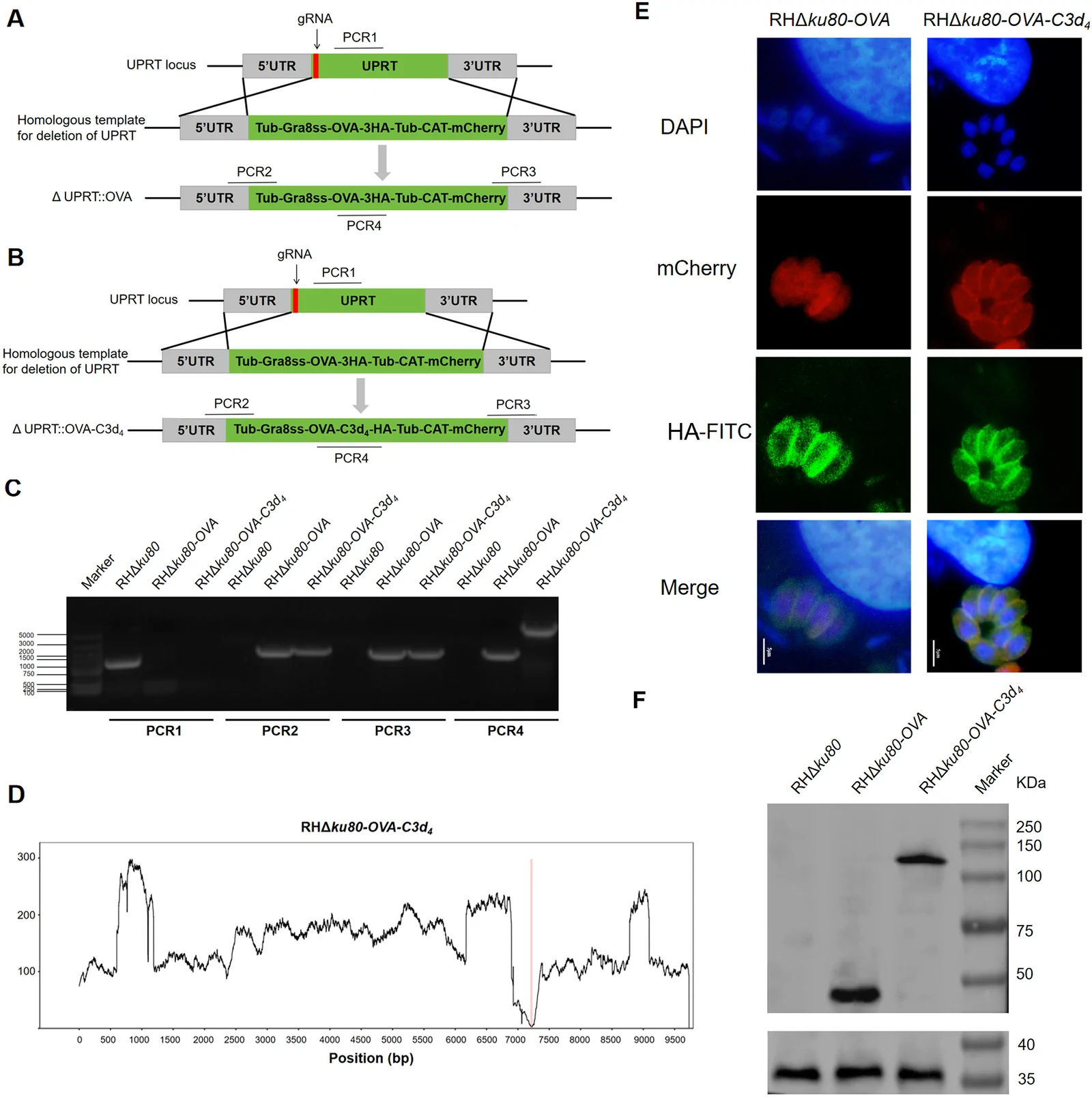
Construction and validation of recombinant *T. gondii* strains expressing OVA and OVA-C3d_4_. **(A)** Schematic of CRISPR/Cas9-mediated replacement of the UPRT locus with the OVA expression cassette in the RHΔ*ku80* strain. The targeting construct contains homology arms flanking the Tub-GRA8ss-OVA-3HA-Tub-mCherry cassette for site-specific integration. **(B)** Schematic of CRISPR/Cas9-mediated integration of the OVA-C3d_4_ expression cassette into the UPRT locus. The construct includes Tub-GRA8ss-OVA-C3d_4_-3HA-mCherry, enabling expression of the OVA-C3d_4_ fusion protein. **(C)** PCR validation of transgenic parasites. PCR1 and PCR2 confirm correct 5′ and 3′ integration of the expression cassette, respectively. PCR3 verifies disruption of the endogenous UPRT locus. PCR4 confirms transgene insertion in RHΔ*ku80-OVA* and RHΔ*ku80-OVA-C3d_4_* strains. **(D)** Whole-genome resequencing coverage analysis of the RHΔ*ku80-OVA-C3d_4_* strain. A localized reduction in read coverage was observed within the tandemly repeated C3d region. Given the high sequence homology among the repeated C3d sequences, this local coverage variation may reflect limitations in short-read mapping across repetitive regions and was therefore not interpreted as definitive evidence of a deletion. Importantly, PCR4 spanning the GRA8SS-OVA-C3d_4_-HA region generated a 5037 bp product consistent with the expected size of the designed construct. **(E)** Immunofluorescence assay (IFA) showing expression and localization of recombinant proteins in intracellular tachyzoites. Nuclei are stained with DAPI (blue), mCherry-expressing parasites are shown in red, and recombinant fusion protein was detected via an anti-HA primary antibody followed by an FITC-conjugated secondary antibody (green). Merged images show localization of OVA within the parasitophorous vacuole. Scale bars, 5 μm. **(F)** Western blot analysis of recombinant proteins in RHΔ*ku80*, RHΔ*ku80-OVA*, and RHΔ*ku80-OVA-C3d_4_* tachyzoites using an anti-OVA antibody. GAPDH was used as a loading control.

Together, these results confirm the successful construction of recombinant *T. gondii* strains with stable integration and expression of OVA and OVA-C3d_4_.

### 3.2 OVA or OVA-C3d_4_ expression does not affect parasite fitness or virulence

To assess the impact of OVA or OVA-C3d_4_ expression on parasite phenotypes, both *in vitro* and *in vivo* assays were performed. In the mouse infection model, survival rates were comparable among mice infected with RHΔ*ku80*, RHΔ*ku80-OVA*, or RHΔ*ku80-OVA-C3d_4_*. All infected animals succumbed within a similar time frame, indicating unchanged acute virulence (**Figure 2A**).

**Figure 2:**
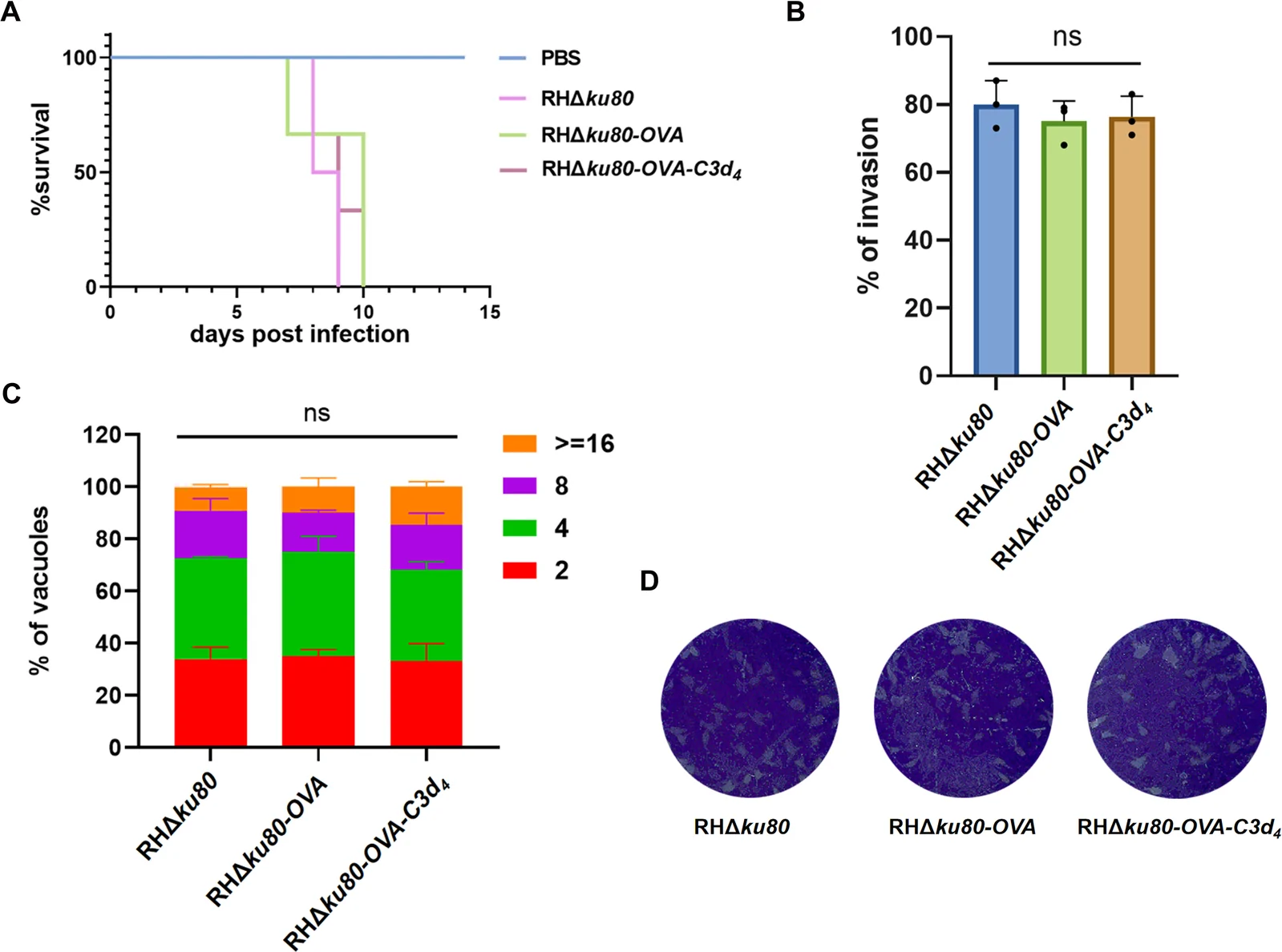
Expression of OVA or OVA-C3d_4_ does not affect parasite fitness or virulence. **(A)** Survival of mice infected with the indicated *T. gondii* strains. Survival curves are monitored over time and analyzed using the Gehan–Breslow–Wilcoxon test; no significant differences are observed (P > 0.05, n = 5 per group). **(B)** Quantification of invasion efficiency of the indicated strains in HFF cells. Data represent mean ± SD from three independent experiments (one-way ANOVA with Tukey’s multiple comparisons test: RHΔ*ku80* vs RHΔ*ku80-OVA*, p=0.6288; RHΔ*ku80* vs RHΔ*ku80-OVA-C3d_4_*, p=0.7721; RHΔk*u80-OVA* vs RHΔ*ku80-OVA-C3d_4_*, p=0.9651; overall ANOVA p=0.6356). ns, not significant. **(C)** Quantification of intracellular replication of the indicated strains in HFF monolayers at 24 h post-infection. Parasite numbers per parasitophorous vacuole were quantified (at least 100 vacuoles per condition, three independent replicates; one-way ANOVA: vacuoles containing 2 parasites, p=0.9173; 4 parasites, p=0.3391; 8 parasites, p=0.5795; ≥16 parasites, p=0.0580). ns, not significant. **(D)** Representative images of plaques formed by the indicated strains in HFF monolayers after 7 days, showing comparable plaque size and number among groups.

In the *in vitro* invasion assay, invasion efficiency did not differ among the groups, suggesting that expression of OVA or OVA-C3d_4_ does not impair host cell entry (**Figure 2B**). Similarly, intracellular replication assays indicated that all strains exhibited comparable proliferative capacity, as evidenced by similar distributions of parasite numbers per parasitophorous vacuole, with no significant differences detected (**Figure 2C**).

Plaque assays further showed comparable plaque morphology and numbers among the three strains, indicating that the parasite phenotype was not affected (**Figure 2D**).

Together, these results indicate that expression of OVA or OVA-C3d_4_ does not significantly affect parasite growth or virulence, supporting their application in subsequent immunological studies.

### 3.3 Induction of potent OVA-specific IFN-γ-mediated cellular immunity by OVA-expressing *T. gondii* live vaccine vectors

Antigen-specific cellular immune responses induced by the recombinant strains were evaluated by measuring IFN-γ production using ELISPOT after stimulation with OVA-derived peptides (OVA257–264 and OVA323–339). As shown in **Figures 3B–E**, mice immunized with OVA protein, RHΔ*ku80-OVA*, or RHΔ*ku80-OVA-C3d_4_* exhibited significantly higher numbers of IFN-γ–secreting cells than those in the unimmunized control (UUC) and RHΔ*ku80* groups. Notably, the RHΔ*ku80-OVA-C3d_4_* group showed a trend toward higher IFN-γ responses than the RHΔ*ku80-OVA* group; however, this difference was not statistically significant (OVA 257-264: p=0.1783, ns; OVA 323-339: p=0.2843, ns). In contrast, mice immunized with the parental RHΔ*ku80* strain exhibited minimal responses, which were comparable to those of the UUC group. These trends were observed for both the CD8^+^ T cell–associated epitope (OVA 257-264) and the CD4^+^ T cell–associated epitope (OVA 323-339), indicating that OVA expression effectively induces antigen-specific cellular immunity. Although the C3d_4_-containing recombinant strains showed a tendency toward higher IFN-γ/cellular responses, these differences were not statistically significant compared with RHΔ*ku80-OVA*. Collectively, these results demonstrate that recombinant *T. gondii* expressing OVA, particularly when fused with C3d_4_, elicits robust OVA-specific IFN-γ responses, supporting its potential as a live vector for the induction of cellular immunity.

**Figure 3:**
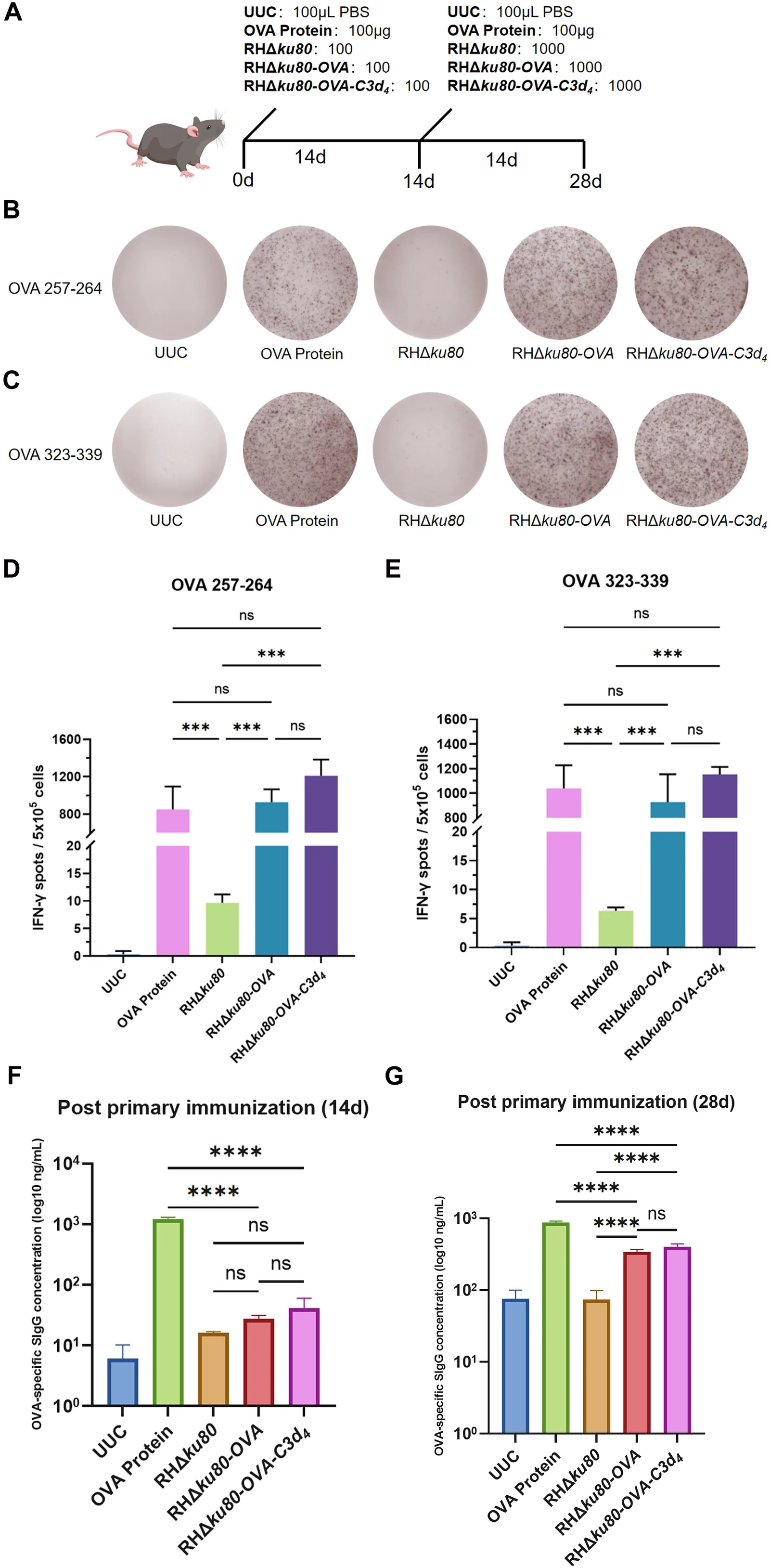
Recombinant *T. gondii* expressing OVA-C3d_4_ enhances antigen-specific cellular and humoral immune responses. **(A)** Schematic representation of the immunization and sampling schedule. Mice were immunized on day 0 and boosted on day 14 with PBS, OVA protein, or the indicated *T. gondii* strains. Samples were collected on day 28 for analysis. Drinking water containing sulfadiazine sodium (400 mg/L) was provided beginning 72 h after each parasite inoculation to control acute parasite replication. **(B, C)** Representative ELISPOT images showing IFN-γ–secreting cells from splenocytes stimulated with OVA-derived peptides OVA257–264 **(B)** and OVA323–339 **(C)**. Each spot represents a single IFN-γ–producing cell. **(D, E)** Quantification of IFN-γ–producing cells in response to stimulation with OVA257–264 **(D)** and OVA323–339 **(E)** (n = 5 mice per group; one-way ANOVA with Tukey’s multiple comparisons test). **(D)** UUC vs OVA Protein, p=0.0001; OVA Protein vs RHΔ*ku80*, p=0.0001; RHΔ*ku80* vs RHΔ*ku80-OVA*, p<0.0001; OVA Protein vs RHΔ*ku80-OVA*, p=0.9774 (ns); OVA Protein vs RHΔ*ku80-OVA-C3d_4_*, p=0.0572 (ns); RHΔ*ku80-OVA* vs RHΔ*ku80-OVA-C3d_4_*, p=0.1783 (ns). **(E)** UUC vs OVA Protein, p<0.0001; OVA Protein vs RHΔ*ku80*, p<0.0001; RHΔ*ku80* vs RHΔ*ku80-OVA*, p<0.0001; OVA Protein vs RHΔ*ku80-OVA*, p=0.8625 (ns); OVA Protein vs RHΔ*ku80-OVA-C3d_4_*, p=0.8611 (ns); RHΔ*ku80-OVA* vs RHΔ*ku80-OVA-C3d_4_*, p=0.2843 (ns). **(F, G)** Serum OVA-specific IgG measured by ELISA at day 14 **(F)** or day 28 **(G)** post-primary immunization (n = 5 mice per group; one-way ANOVA with Tukey’s multiple comparisons test). **(F)** OVA Protein vs RHΔ*ku80-OVA*, p<0.0001; OVA Protein vs RHΔ*ku80-OVA-C3d_4_*, p<0.0001; RHΔ*ku80* vs RHΔ*ku80-OVA*, p=0.9975 (ns); RHΔ*ku80* vs RHΔ*ku80-OVA-C3d_4_*, p=0.9586 (ns); RHΔ*ku80-OVA* vs RHΔ*ku80-OVA-C3d_4_*, p=0.9960 (ns). **(G)** OVA Protein vs RHΔ*ku80-OVA*, p<0.0001; OVA Protein vs RHΔ*ku80-OVA-C3d_4_*, p<0.0001; RHΔ*ku80* vs RHΔ*ku80-OVA*, p<0.0001; RHΔ*ku80* vs RHΔ*ku80-OVA-C3d_4_*, p<0.0001; RHΔ*ku80-OVA* vs RHΔ*ku80-OVA-C3d_4_*, p=0.2541 (ns).

### 3.4 Fusion with C3d_4_ modulates antibody isotype responses following immunization

To further evaluate the effect of antigen dose and C3d_4_ fusion on humoral immune polarization, mice were immunized with increasing doses of RHΔ*ku80-OVA* or RHΔ*ku80-OVA-C3d_4_* strains, and antibody isotype responses were assessed by measuring the IgG1/IgG2a ratio (**Figure 4A**). As shown in **Figure 4B**, immunization with OVA protein alone resulted in a relatively low IgG1/IgG2a ratio.

**Figure 4:**
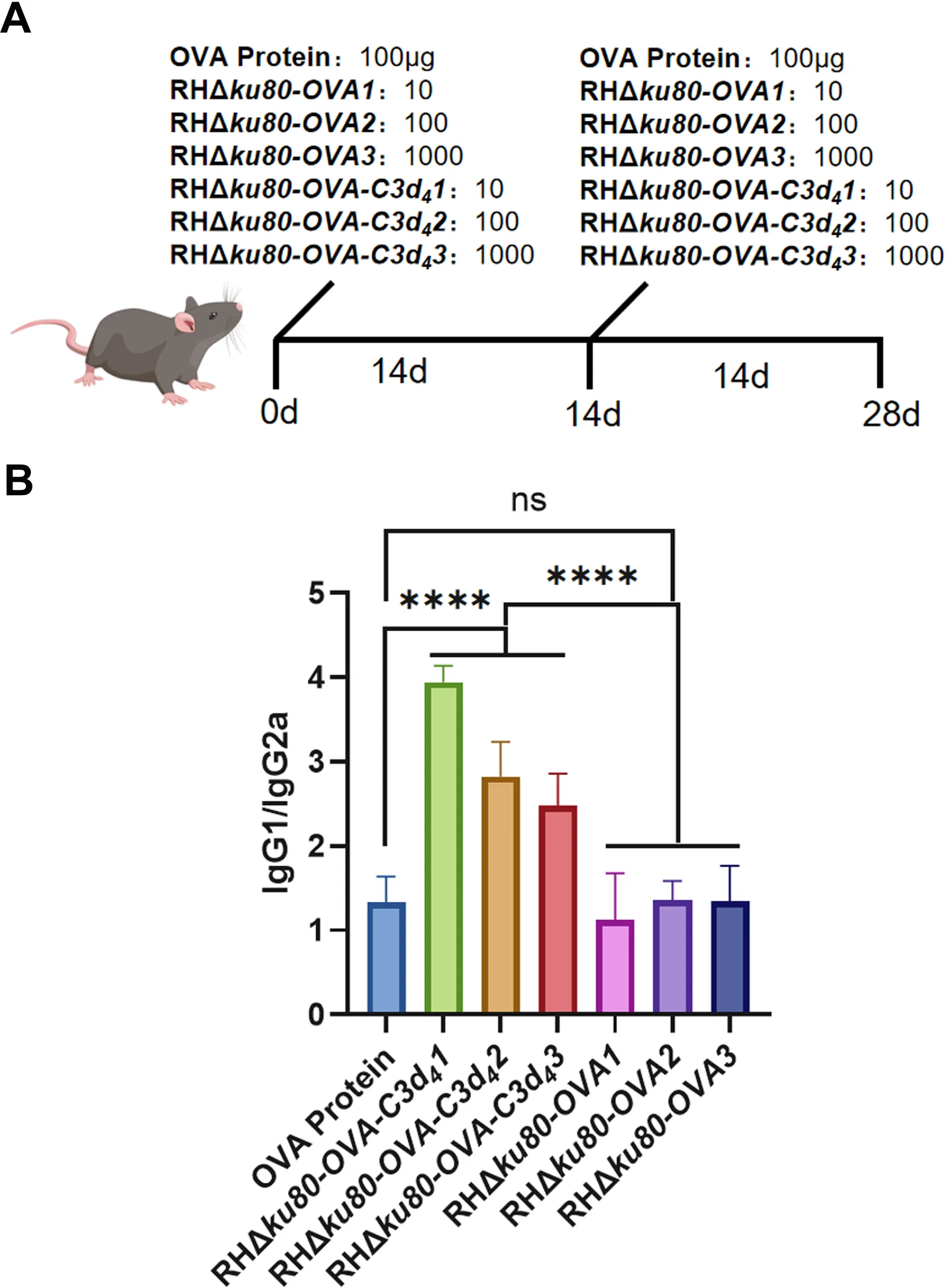
C3d_4_ fusion modulates antibody isotype responses in a dose-dependent manner. **(A)** Schematic diagram of the immunization schedule. Mice were immunized at day 0 and boosted at day 14 with OVA protein (100 μg) or the indicated doses (10, 100, or 1000 tachyzoites) of RHΔ*ku80-OVA* or RHΔ*ku80-OVA-C3d_4_* strains. Samples were collected at day 28 for analysis. **(B)** Serum antibody isotype responses measured as the IgG1/IgG2a ratio. Mice immunized with RHΔ*ku80-OVA-C3d_4_* exhibited altered IgG1/IgG2a ratios compared with RHΔ*ku80-OVA* across different doses.(n = 10 mice per group; one-way ANOVA with Tukey’s multiple comparisons test): RHΔ*ku80-OVA-C3d_4_* (10) vs RHΔ*ku80-OVA* (10), p<0.0001; RHΔ*ku80-OVA-C3d_4_* (100) vs RHΔ*ku80-OVA* (100), p=0.0001; RHΔ*ku80-OVA-C3d_4_* (1000) vs RHΔ*ku80-OVA* (1000), p=0.0003; among the RHΔ*ku80-OVA* dose groups, p=0.3409-0.9988 (ns); OVA Protein vs RHΔ*ku80-OVA-C3d_4_* (10), p<0.0001. ns, not significant; ****p<0.0001.

In contrast, mice immunized with RHΔ*ku80-OVA-C3d_4_* exhibited markedly elevated IgG1/IgG2a ratios across all tested doses, with the highest response observed at the lowest immunization dose. This trend was not evident in mice immunized with RHΔ*ku80-OVA*, which showed comparatively lower and more stable IgG1/IgG2a ratios regardless of dose.

Statistical analysis revealed that the IgG1/IgG2a ratios in the RHΔ*ku80-OVA-C3d_4_* groups were significantly higher than those in the corresponding RHΔ*ku80-OVA* groups at equivalent doses (P < 0.0001) whereas no significant differences were observed among the RHΔ*ku80-OVA* groups themselves (P > 0.05).

Together with the ELISA results obtained under the standard immunization regimen (**Figures 3F, G**), these findings suggest that fusion with C3d_4_ modulates the qualitative nature of the antibody response rather than substantially increasing overall antibody titers. Specifically, the increased IgG1/IgG2a ratio indicates a relative predominance of IgG1 over IgG2a in the C3d_4_-containing groups.

### 3.5 OVA-expressing *T. gondii* elicits antigen-specific protection against EfOVA

To evaluate the protective efficacy of recombinant parasites, mice were immunized with RHΔ*ku80*, RHΔ*ku80-OVA*, or RHΔ*ku80-OVA-C3d_4_* strains and subsequently challenged with EfOVA (**Figure 5A**).

**Figure 5:**
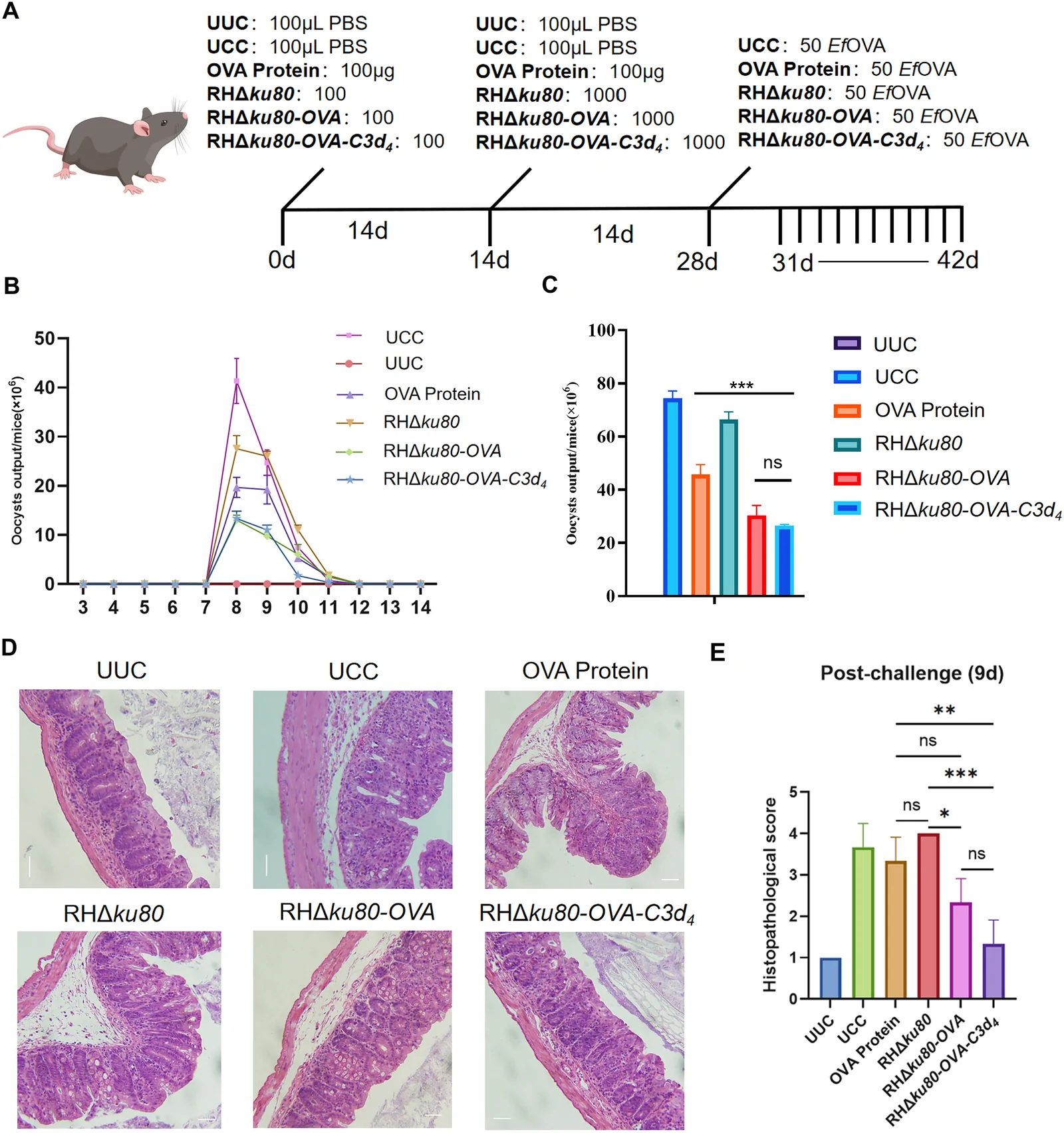
Immunization with OVA-expressing *T. gondii* reduces oocyst shedding and intestinal pathology following antigen-matched *Eimeria* challenge. **(A)** Experimental design and immunization schedule. Mice were immunized with PBS (UUC), PBS followed by challenge (UCC), OVA protein, RHΔ*ku80*, RHΔ*ku80-OVA*, or RHΔ*ku80-OVA-C3d_4_* at day 0 and boosted at day 14. At day 28, mice were challenged with OVA-expressing *Eimeria* oocysts, and samples were collected as indicated. **(B)** Kinetics of fecal oocyst shedding following challenge. Oocyst output was monitored daily from day 3 to day 14 post-challenge, (n = 5 mice/strain). **(C)** Quantification of total oocyst output. Data are presented as cumulative oocyst counts five mice. Quantification of total oocyst output per mouse (one-way ANOVA with Tukey’s multiple comparisons test): UCC vs RHΔ*ku80-OVA*, p<0.0001; UCC vs RHΔk*u80-OVA-C3d_4_*, p<0.0001; UCC vs OVA Protein, p<0.0001; RHΔ*ku80-OVA* vs RHΔ*ku80-OVA-C3d_4_*, p=0.5310 (ns). **(D)** Histopathological analysis of cecal tissues at 9 days post-challenge. Representative hematoxylin and eosin (H&E)-stained sections are shown. Severe mucosal damage and inflammatory infiltration were observed in the UCC group, whereas reduced lesions were evident in mice immunized with RHΔ*ku80-OVA* or RHΔ*ku80-OVA-C3d_4_*. Scale bars, 40 μm. **(E)** Histopathological scoring of cecal lesions. Lesion severity was evaluated based on epithelial damage and inflammatory infiltration. Histopathological lesions were as described in Methods. (one-way ANOVA with Tukey’s multiple comparisons test): OVA Protein vs RHΔ*ku80-OVA-C3d_4_*, p=0.0024; OVA Protein vs RHΔ*ku80-OVA*, p=0.1711 (ns); OVA Protein vs RHΔ*ku80*, p=0.5380 (ns); RHΔ*ku80* vs RHΔ*ku80-OVA*, p=0.0100; RHΔ*ku80* vs RHΔ*ku80-OVA-C3d_4_*, p=0.0002; RHΔ*ku80-OVA* vs RHΔ*ku80-OVA-C3d_4_*, p=0.1711 (ns).

Fecal oocyst output was monitored daily after challenge. As shown in **Figure 5B**, mice in the unimmunized challenged control (UCC) group exhibited a sharp peak of oocyst shedding at 8–9 days post-challenge. In contrast, mice immunized with RHΔ*ku80-OVA* or RHΔ*ku80-OVA-C3d_4_* displayed markedly reduced oocyst excretion, with both peak intensity and overall shedding significantly decreased. Immunization with the parental RHΔ*ku80* strain or OVA protein alone resulted in only limited or moderate reduction. Quantitative analysis confirmed that oocyst output in the RHΔ*ku80-OVA* and RHΔ*ku80-OVA-C3d_4_* groups was significantly lower than that in the UCC group (**Figure 5C**), whereas no significant difference was observed between the two recombinant strains (p=0.5310, ns).

Histopathological examination of the cecum at 9 days post-challenge further supported these findings (**Figure 5D**). Severe epithelial disruption, inflammatory cell infiltration, and mucosal damage were observed in the UCC group. In contrast, mice immunized with RHΔ*ku80-OVA* or RHΔ*ku80-OVA-C3d_4_* showed notably preserved epithelial architecture and reduced inflammatory infiltration.

Collectively, these results demonstrate that immunization with OVA-expressing *T. gondii* confers significant protection against antigen-matched *Eimeria* infection, as evidenced by reduced parasite replication and alleviated intestinal pathology. The inclusion of C3d_4_ did not result in a statistically significant enhancement of protection under the conditions tested.

## 4 Discussion

In this study, we demonstrated that genetically engineered *T. gondii* can function as an effective live vector for heterologous antigen delivery and induction of OVA-specific immune responses that protected against OVA-expressing *E. falciformis* challenge. Using recombinant RHΔ*ku80* strains expressing OVA alone or fused with multiple copies of C3d_4_, we systematically evaluated transgene stability, parasite fitness, immunogenicity, and protective efficacy in a murine infection model. Our findings support three principal conclusions: (i) recombinant *T. gondii* stably expresses heterologous antigens without compromising parasite fitness or virulence; (ii) OVA expression induces strong antigen-specific cellular immune responses associated with IFN-γ production; and (iii) although C3d_4_ modulates certain immunological parameters, it does not significantly enhance protection against *Eimeria* challenge.

A critical requirement for live-vector vaccine development is preservation of vector fitness following genetic manipulation (18). In the present study, insertion of OVA or OVA-C3d_4_ into the UPRT locus had no detectable effects on parasite invasion, intracellular replication, plaque formation, or acute virulence in mice. These observations are consistent with previous studies using RHΔ*ku80-derived* strains, in which targeted integration into non-essential loci generally maintains parasite viability and biological characteristics (19–21). The stable phenotype observed here further supports the suitability of *T. gondii* as a robust platform for sustained antigen expression *in vivo* (9, 22, 23).

A key consideration in the design of the recombinant strains was the subcellular localization of the heterologous antigen. In the present study, the GRA8 signal sequence was used to direct OVA and OVA-C3d_4_ into the dense-granule-associated secretory pathway and PV-associated compartment. This strategy was selected to place the heterologous antigen within the intracellular environment generated during parasite infection, thereby potentially facilitating its access to host antigen-processing pathways and supporting the induction of antigen-specific cellular immune responses. The use of GRA8-derived targeting signals is supported by previous studies in apicomplexan parasites showing that the *T. gondii* GRA8 signal sequence can direct reporter proteins to the PV of transfected *Eimeria tenella*, suggesting functional conservation of this targeting mechanism across related parasites (24, 25).

We did not directly compare dense-granule/PV targeting with alternative antigen-localization strategies, such as parasite surface expression through GPI anchoring or fusion to surface antigens. Different subcellular localizations may influence antigen accessibility, processing, and the resulting balance between humoral and cellular immune responses. Therefore, systematic comparisons of antigen localization strategies will be important for optimizing recombinant apicomplexan parasites as vaccine delivery platforms.

Immunological analyses demonstrated that *T. gondii-mediated* antigen delivery efficiently induced antigen-specific cellular immune responses (26–28). Immunization with recombinant parasites significantly increased IFN-γ–secreting splenocytes in response to both CD4^+^ and CD8^+^ OVA epitopes, indicating effective antigen processing and presentation during intracellular infection. These findings are consistent with the established capacity of *T. gondii* to activate dendritic cells and promote IL-12–dependent immune polarization, which plays a central role in host defense against intracellular pathogens, including coccidia (29). Importantly, parental RHΔ*ku80* parasites elicited minimal OVA-specific responses, demonstrating that the observed protection was primarily antigen dependent rather than the result of nonspecific immune activation (30). However, the specific cellular sources of IFN-γ and the contributions of CD4^+^ and CD8^+^ T cells to protection were not determined and require further investigation.

C3d_4_ was incorporated as a molecular adjuvant to enhance antigen immunogenicity through engagement of complement receptor 2 (CR2/CD21), a strategy previously reported to augment humoral responses in multiple vaccine systems (13, 31–33). In our model, fusion with C3d_4_ modestly altered antibody isotype distribution and slightly increased IFN-γ–associated responses. However, these immunological differences did not translate into a statistically significant additional protective benefit of the C3d_4_-containing recombinant strain compared with the OVA-expressing strain following *Eimeria* challenge. One possible explanation is that immunity against *Eimeria* is predominantly mediated by cellular rather than humoral immune mechanisms, thereby limiting the contribution of B cell–oriented adjuvant effects (34). In addition, the intrinsically strong immunostimulatory properties of *T. gondii* may already maximize antigen presentation and inflammatory signaling, potentially masking the additive effect of C3d_4_ (35). Notably, whole-genome resequencing revealed reduced coverage within part of the repeated C3d_4_ region. Although the recombinant strain was validated by PCR and Western blotting, the precise integrity and functional contribution of the C3d repeats require further investigation. The present findings should therefore be interpreted cautiously, as no significant additional protective benefit was observed for the C3d_4_-containing recombinant strain under the conditions tested.

Challenge experiments demonstrated that immunization with OVA-expressing *T. gondii* significantly reduced oocyst shedding and alleviated intestinal pathology following antigen-matched *Eimeria* infection. These findings provide direct evidence that antigen-specific immunity induced by a heterologous protozoan vector can mediate cross-species protection when a shared antigen is targeted. Notably, the absence of significant differences between RHΔ*ku80-OVA* and RHΔ*ku80-OVA-C3d_4_* in both parasite burden and histopathological scores suggests that the C3d_4_-mediated changes in antibody isotype distribution were not associated with additional protective efficacy, while the observed antigen-specific cellular immune responses may contribute importantly to protection in this model.

Collectively, our findings support the concept that *T. gondii* can serve as a versatile protozoan vaccine platform capable of inducing protective antigen-specific immunity against coccidial infection. This strategy offers several advantages, including efficient intracellular antigen delivery, strong intrinsic immunostimulatory activity, and stable genomic integration. At the same time, the limited additive effect of C3d_4_ highlights an important consideration for vaccine design: adjuvants that primarily enhance humoral immunity may provide limited benefit in infections predominantly controlled by T cell–mediated mechanisms (36).

Several limitations should also be acknowledged. First, OVA was used as a model antigen and does not fully reflect the complexity of native protective antigens derived from *Eimeria*. Future studies should evaluate parasite-derived antigens to better assess translational potential. Second, the mechanistic basis of protection, including tissue-resident immunity, T-cell subset dynamics, and cytokine networks, was not extensively investigated. Furthermore, because *Eimeria* infection occurs in the intestinal mucosa, systemic splenic IFN-γ responses may not fully reflect protective immune mechanisms at the site of infection. The present study did not characterize intraepithelial or lamina propria lymphocyte responses or tissue-resident memory T-cell populations. Future studies should therefore investigate intestinal immune responses, including tissue-resident T-cell phenotypes and local cytokine production, to better define the mucosal mechanisms associated with protection. Finally, the durability of immune memory and the extent of heterologous protection against other *Eimeria* species remain unclear and warrant further study.

In conclusion, this work demonstrates that recombinant *T. gondii* expressing heterologous antigens can induce robust antigen-specific cellular immunity and confer partial protection against *Eimeria* infection. Although C3d_4_ modulates aspects of immune polarization, its contribution to protective efficacy appears limited in this predominantly cell-mediated infection model. These findings provide important insights into protozoan-based vaccine vector design and further highlight the potential contribution of antigen-specific cellular immunity to protection against coccidiosis.

## Data Availability

The datasets presented in this study can be found in online repositories. The names of the repository/repositories and accession number(s) can be found in the article/**Supplementary Material**.
