# Supplementary material for: Recombinant Toxoplasma gondii expressing OVA confers antigen-specific protection against Eimeria falciformis
Source: Front Immunol. 2026 Sep 16;17:1915667. doi: 10.3389/fimmu.2026.1915667 (PMC13624080; doi:10.3389/fimmu.2026.1915667)
Supplement: Supplementary file 1 [file Table1.docx]

Supplementary Material

# 1 Supplementary Data

The optimized C3d sequence

ACGCCGGCGGGCTGCGGCGAGCAGCACATGATCGGCATGACGCCGACGGTCATCGCGGTCCACTACCTCGACCAGACGGAGCAGTGGGGCAAGTTCGGCATCGAGAAGCGCCAGGAGGCGCTCGAGCTCATCAAGAAGGGCTACACGCAGCAGCTCGCGTTCAAGCAGCCGTCTTCTGCGTACGCGGCGTTCAACAACCGCCCGCCGTCTACGTGGCTCACGGCGTACGTCGTCAAGGTCTTCTCTCTCGCGGCGAACCTCATCGCGATCGACTCTCACGTCCTCTGCGGCGCGGTCAAGTGGCTCATCCTCGAGAAGCAGAAGCCGGACGGCGTCTTCCAGGAGGACGGCCCGGTCATCCACCAGGAGATGATCGGCGGCTTCCGCAACGCGAAGGAGGCGGACGTCTCTCTCACGGCGTTCGTCCTCATCGCGCTCCAGGAGGCGCGCGACATCTGCGAGGGCCAGGTCAACTCTCTCCCGGGCTCTATCAACAAGGCGGGCGAGTACATCGAGGCGTCTTACATGAACCTCCAGCGCCCGTACACGGTCGCGATCGCGGGCTACGCGCTCGCGCTCATGAACAAGCTCGAGGAGCCGTACCTCGGCAAGTTCCTCAACACGGCGAAGGACCGCAACCGCTGGGAGGAGCCGGACCAGCAGCTCTACAACGTCGAGGCGACGTCTTACGCGCTCCTCGCGCTCCTCCTCCTCAAGGACTTCGACTCTGTCCCGCCGGTCGTCCGCTGGCTCAACGAGCAGCGCTACTACGGCGGCGGCTACGGCTCTACGCAGGCGACGTTCATGGTCTTCCAGGCGCTCGCGCAGTACCAGACGGACGTCCCGGACCACAAGGACCTCAACATGGACGTCTCTTTCCACCTCCCGTCTAGATCC

# 2 Supplementary Tables

**Table S1.** Primers used for PCR identification of the RHΔ*ku80-OVA* and RHΔ*ku80-OVA-C3d₄* strains.

| Primers | Sequence（5’ to 3’） | Length (bp) |
| --- | --- | --- |
| PCR1-F | TTCCCAATGTGGTGCTCATGAAG | 316 |
| PCR1-R | AATTCTTCCTTCGGTGTTTCTTTA |  |
| PCR2-F | CTTGGGGATGACCCTTGTCG | 1406 |
| PCR2-R | GCGATCGGGCAGTAGAAGAT |  |
| PCR3-F | TCAAGCAGAGGCTGAAGCTG | 1343 |
| PCR3-R | CACTGTTCAATCCCCACCGT |  |
| PCR4-F | ATGGCTTTACCATTGCGTGT | 1333/5037 |
| PCR4-R | AAGCGTAATCTGGAACGTC |  |

**Table S2.** Experimental design for animal experiments (Immunization Protocol 1)

| Groups | Numbers of mice | Primary immunization per mouse | Second immunization  per mouse | Challenge  (50 EfOVA) |
| --- | --- | --- | --- | --- |
| UUC | 15 | / | / | / |
| UCC | 15 | / | / | √ |
| OVA protein | 15 | 100 μg | 100 μg | √ |
| RHΔ*ku80* | 15 | 100 tachyzoites | 1000 tachyzoites | √ |
| RHΔ*ku80-OVA* | 15 | 100 tachyzoites | 1000 tachyzoites | √ |
| RHΔ*ku80-OVA-C3d₄* | 15 | 100 tachyzoites | 1000 tachyzoites | √ |

**Table S3.** Experimental design for animal experiments (Immunization Protocol 2)

| Groups | Numbers of mice | Primary immunization per mouse | Second immunization  per mouse |
| --- | --- | --- | --- |
| OVA protein | 10 | 100 μg | 100 μg |
| RHΔ*ku80-OVA1* | 10 | 10 tachyzoites | 10 tachyzoites |
| RHΔ*ku80-OVA2* | 10 | 100 tachyzoites | 100 tachyzoites |
| RHΔ*ku80-OVA3* | 10 | 1000 tachyzoites | 1000 tachyzoites |
| RHΔ*ku80-OVA-C3d₄1* | 10 | 10 tachyzoites | 10 tachyzoites |
| RHΔ*ku80-OVA-C3d₄2* | 10 | 100 tachyzoites | 100 tachyzoites |
| RHΔ*ku80-OVA-C3d₄3* | 10 | 1000 tachyzoites | 1000 tachyzoites |
